# Histologic and Morphometric Comparison of Drusen Associated With Chronic Retinal Detachment and Aging-Associated Drusen

**DOI:** 10.1167/tvst.15.1.32

**Published:** 2026-01-27

**Authors:** Arina Nisanova, Jonathan H. Lin, Rachel L. Frauches, Martin Estrada, Andrew J. Nelson

**Affiliations:** 1 Department of Ophthalmology, Baylor College of Medicine, Houston, TX, USA; 2 Department of Ophthalmology and Vision Science, University of California – Davis, Sacramento, CA, USA; 3 Department of Pathology, Stanford University, Stanford, CA, USA; 4 Department of Ophthalmology, Stanford University, Stanford, CA, USA; 5 Veterans Affairs, Palo Alto, CA, USA

**Keywords:** drusen, large drusen, drusen, retinal detachment (RD), young, trauma, injury

## Abstract

**Purpose:**

Drusen are subretinal pigment epithelium extracellular matrix deposits. This study compared histomorphometric characteristics of age-related and atypical drusen found in an eye of a young woman with chronic retinal detachment (RD).

**Methods:**

We conducted a histomorphometric examination of drusen identified in 3 eyes: an eviscerated eye from a 35-year-old woman with chronic RD, and 2 specimens of 2 patients, aged 91 and 77 years, with typical age-related drusen. Sections of formalin-fixed tissue stained with hematoxylin and eosin (H&E), periodic acid-Schiff (PAS), Masson trichrome, luxol fast blue (LFB), and Von Kossa were evaluated using light microscopy. Drusen dimensions were quantified using Philips Digital Pathology Software.

**Results:**

Atypical drusen identified in the RD eye (*n* = 25) displayed spheroidal shape with peripherally radiating fibrils. There were foci of dystrophic calcification and localized chronic choroidal inflammation and within some drusen. Age-related drusen (*n* = 29) had a typical dome-shaped appearance. Atypical drusen had significantly larger (*P* < 0.001) average circumference (278 vs. 52 µm), diameter (96 vs. 20 µm), and area (0.006 vs. 0.00022 mm^2^) than age-related drusen, respectively. Both groups stained positively with PAS, negatively with LFB and Von Kossa, and blue on Masson Trichrome.

**Conclusions:**

Atypical drusen found in this RD case display significant differences in morphometric parameters compared to age-related drusen but similar histochemical staining patterns, suggesting a similar macromolecular composition. Further investigations are needed to inform drusen pathogenesis, morphology, and associations with sex, age, or long-standing RD.

**Translational Relevance:**

This case adds to the limited body of evidence on drusen in younger patients and suggests that atypical drusen may form in association with RD.

## Introduction

First described by Franciscus Donders in 1855 as “Colloidkugeln,”^1^ drusen are extracellular matrix deposits of proteins, lipids, and cellular debris found between the basal surface of the retinal pigment epithelium (RPE) and Bruch's membrane.^2^ Drusen are commonly associated with aging and are a hallmark of age-related macular degeneration (AMD), but they can also manifest in other retinal diseases.^3^ Drusen may be classified by size, shape, location, appearance, and molecular composition.^2^ Different subtypes of clinically detectable deposits include hard, soft, cuticular, reticular, calcified drusen, and pseudodrusen.^2^^,^^4^ Histologically, soft drusen appear as low mounds filled with lipoproteinous debris,^2^^,^^5^ whereas hard drusen are smaller, well-demarcated deposits comprised of homogenous hyaline material.^2^^,^^6^

Over 90% of the population over the age of 50 years is estimated to have at least one druse in one eye,^7^ and small drusen (<63 µm) can be found in 30% to 91.5% of younger adults (aged 18 to 54 years).^8^^,^^9^ Rare case reports described large drusen (>125 µm) found in enucleated eyes with chronic retinal detachment (RD).^10^^,^^11^ However, it is unclear whether they represent a variation of age-related drusen or if drusen formation associated with RD is a distinct pathological entity. Herein, we present a comparison of histologic and morphometric characteristics of age-related drusen with large drusen (>125 µm) found in an eviscerated eye of a young woman with a history of chronic RD and childhood trauma.

## Methods

This is a retrospective study conducted at Stanford University. Three eyes of three subjects were included in the study.

One eye was an evisceration specimen from a 35-year-old woman with chronic RD and phthisis bulbi. The patient had long-standing left eye blindness and underwent evisceration for pain in the eye. The ophthalmic records were scarce. She had undergone an unsuccessful corneal transplantation in the left eye. There was also a long-standing RD in the eye secondary to a traumatic injury during childhood. The ophthalmic examination was notable for a phthisic left eye with esotropic alignment. The visual acuity was 20/20 in the right eye, and she had no light perception in the left eye. No fundus images or optical coherence tomography (OCT) were available for this patient's eviscerated eye due to corneal clouding.

Two specimens of 2 female patients, aged 91 and 77 years, with typical age-related macular and peripheral drusen, were included as controls. The eye of the 77-year-old patient was obtained through a limited autopsy completed for the purpose of Alzheimer's disease research after the patient's death. The patient had no known ocular history, no prior ocular surgeries, and no documented ocular medications. She passed away on hospice from metastatic lung adenocarcinoma. The eye of the 91-year-old patient was obtained through an exenteration for basal cell carcinoma in the left eye. The patient also had a history of corneal scarring from lagophthalmos and compressive optic neuropathy with light perception vision in the exenterated eye.

The eye specimens were fixed in 4% formaldehyde solution after enucleation and evisceration. The eyes were opened in the horizontal axis parallel to the optic nerve and further processed for histopathologic analysis. The sections were dehydrated in alcohol and embedded in paraffin blocks. The slides were stained with hematoxylin and eosin-stained (H&E) for microscopic evaluation. At least 25 macular and peripheral drusen were evaluated from each eye using light microscopy of H&E-stained sections of formalin-fixed tissue. Additional stains were performed with periodic acid-Schiff (PAS), Masson Trichrome, luxol fast blue (LFB), and Von Kossa. Histopathological examination and quantification of drusen circumference, largest diameter, and area were performed using Philips Digital Pathology Software.

The data are reported as a number, percentage, mean, standard deviation (SD), and range, as appropriate. The data were analyzed with a *t*-test with Welch's correction in GraphPad Prism 10 (version 9.5.0, Dotmatics). The findings were considered significant if *P* < 0.05. The study was conducted in accordance with the Declaration of Helsinki. The Institutional Review Board (IRB) at Stanford University approved the study (IRB protocol #54760).

## Results

### Evisceration Specimen

**Figure 1.**
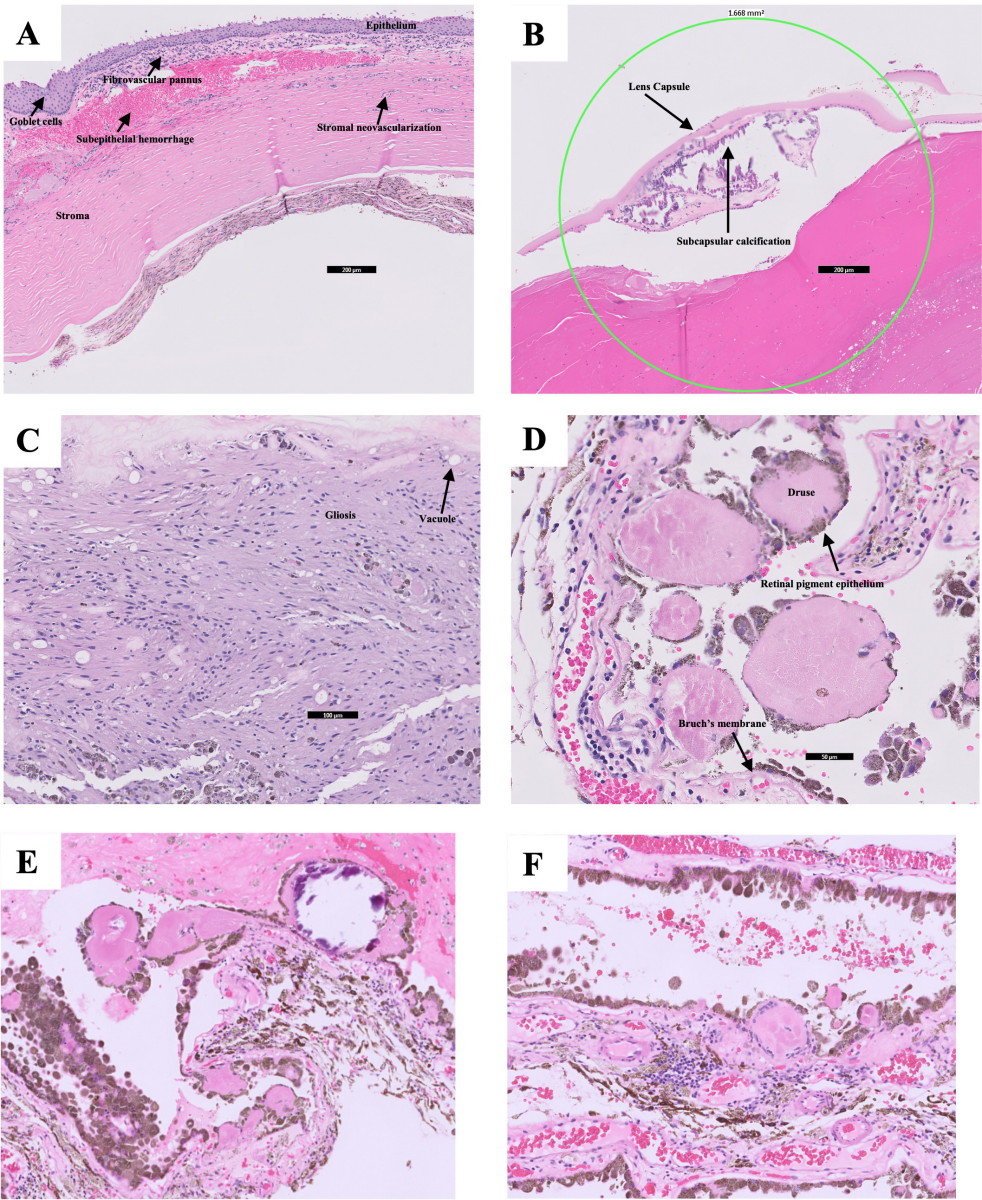
Microscopic examination of the eviscerated eye of a 35-year-old patient with phthisis bulbi and chronic retinal detachment showed: (**A**) cornea with squamous metaplasia with goblet cells, subepithelial fibrovascular pannus with hemorrhage and solar damage, stromal neovascularization, iridocorneal adhesions, and loss of endothelial cells; (**B**) lens with subcapsular calcification and cataract; and (**C**) retina with signs of gliosis and large vacuoles, possibly from the silicone oil. (**D**) Large spheroidal drusen with heterogeneous, amorphous eosinophilic material with peripherally radiating fibrils. (**E**) Foci of dystrophic calcification (crystalline purple deposits) within some drusen. (**F**) Localized chronic choroidal inflammation and inflammation associated with a large druse.

Microscopic examination of the cornea showed squamous metaplasia with goblet cells, subepithelial fibrovascular pannus with hemorrhage and solar damage, stromal neovascularization, iridocorneal adhesions, and loss of endothelial cells (Fig. 1A). The lens was notable for subcapsular calcification and a cataract (Fig. 1B). The retina showed signs of neovascularization, gliosis, and large vacuoles suspected to be intracellular inclusions of silicone oil (Fig. 1C).

Drusenoid deposits were identified between the RPE and Bruch's membrane and were described as spheroidal deposits with heterogeneous, amorphous, eosinophilic material with peripherally radiating fibrils (Fig. 1D). Some drusen had foci of dystrophic calcification (Fig. 1E), and some deposits displayed localized chronic inflammation in the choroid and within a druse (Fig. 1F). Twenty-five drusen were identified for morphometric analysis.

**Figure 2.**
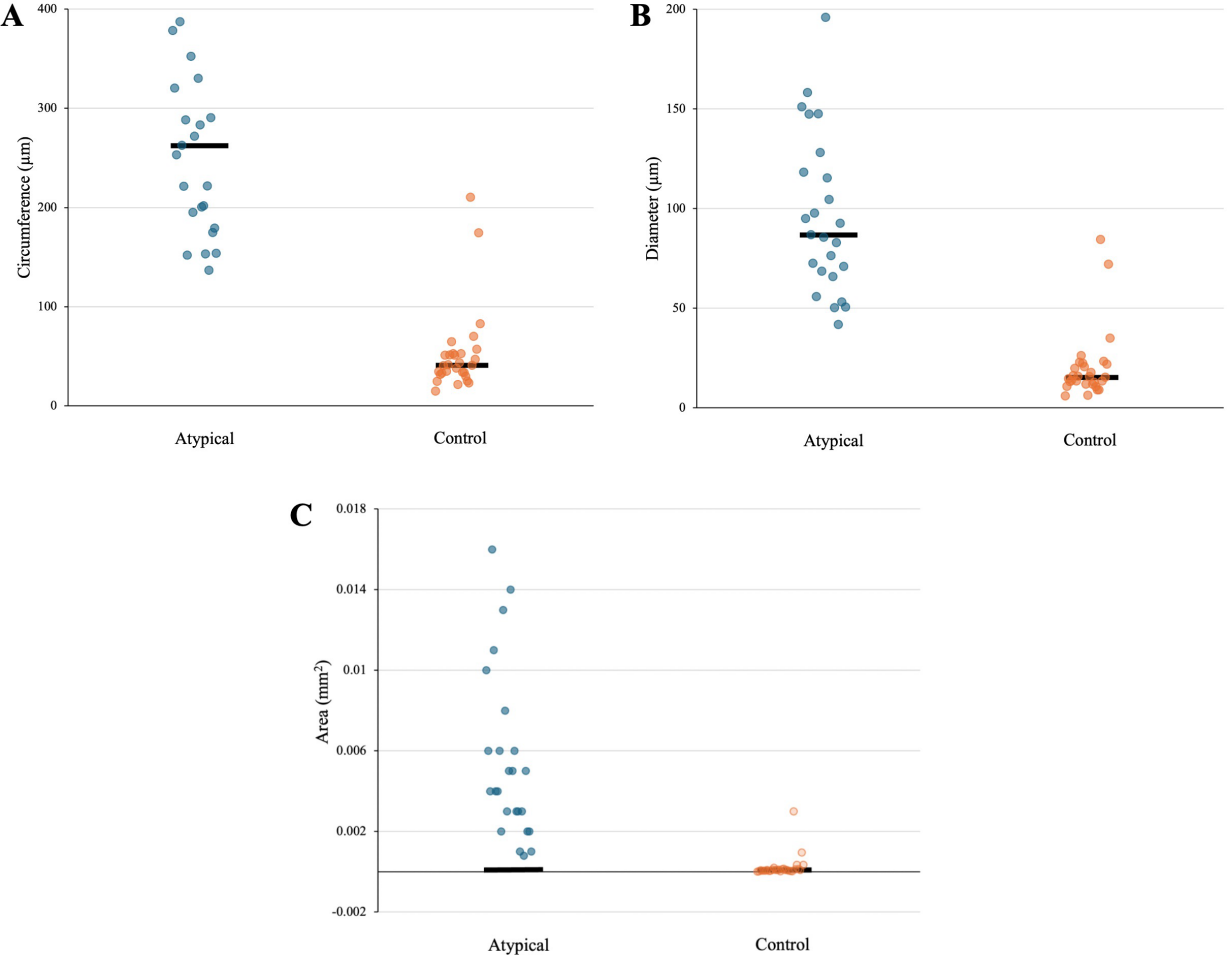
Scatterplot comparison of the morphometric characters illustrates significantly larger circumference (**A**), diameter (**B**), and area (**C**) of atypical compared to control (age-related) drusen. *Black horizontal lines* represent a median.

The average (SD) diameter of these drusen was 96.3 µm (39.9), with the smallest measuring 41.6 µm and the largest 195.7 µm, as shown in the Table and Figure 2. The drusen's average (SD) circumference was 277.5 µm (115.2), ranging from 136.6 µm to 588 µm. The average (SD) area was 0.006 mm^2^ (0.004), ranging from 0.001 to 0.016 mm^2^.

**Table.** Average Dimensions of Atypical (*n* = 25) and Control (*n* = 29) Drusen, Including Circumference (µm), Diameter (µm), and Area (mm^2^) Quantified Using Philips Digital Pathology Software

	Circumference, µm			Diameter, µm			Area, mm^2^		
	Atypical (*n* = 25)	Control (*n* = 29)	*P* Value, Cohen's d	Atypical (*n* = 25)	Control (*n* = 29)	*P* Value, Cohen's d	Atypical (*n* = 25)	Control (*n* = 29)	*P* Value, Cohen's d
Mean	277.5	51.9	<0.001, 2.55	96.3	20	<0.001, 2.68	0.006	0.00009	<0.001
SD	115.2	41.9		39.9	17.4		0.004	0.0006	
Min	136.6	14.6		41.6	5.7		0.001	0.00001	
Max	588	210		195.7	84.3		0.016	0.003	

Atypical drusen displayed significantly larger morphometric parameters than age-related drusen in the control group.

### Eyes With Age-Related Drusen

We identified 29 age-related drusen in the 2 eyes of older patients. These drusen had a characteristic dome-shaped appearance, as seen in AMD,^12^ with occasional retraction artifact. No inflammation or dystrophic calcification was seen.

The average diameter of age-related drusen was 20 µm (17.4), ranging from 5.7 to 84.3 µm. The average (SD) circumference was 51.9 µm (41.9), ranging from 14.6 to 210 µm, and the average (SD) area was 0.00009 mm^2^, with the smallest druse measuring 0.00001 mm^2^ and the largest 0.003 mm^2^ (see Fig. 2).

### Comparison of Histopathologic and Morphometric Findings

**Figure 3.**
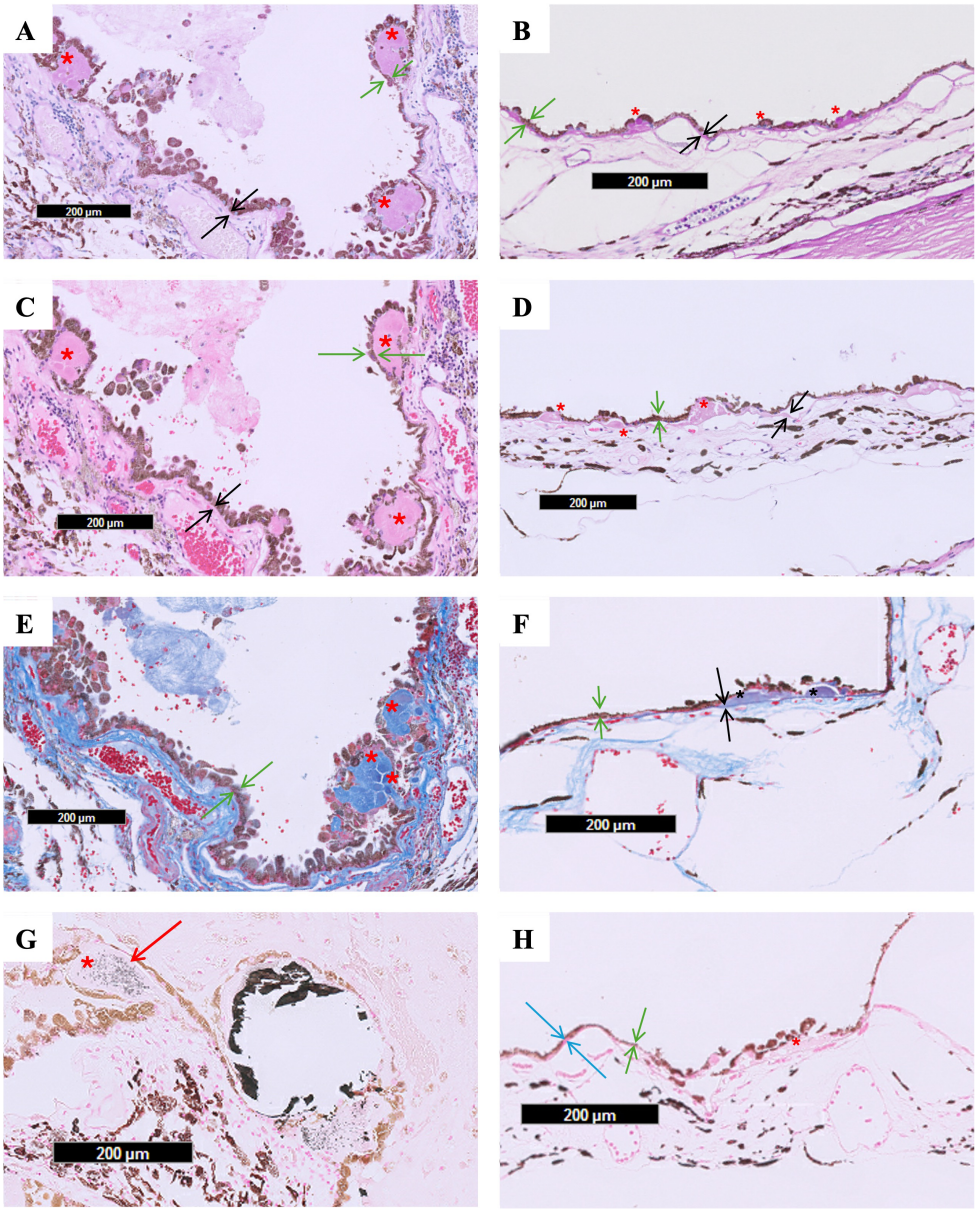
(**A, C, E, G**) Illustrate slides from the eviscerated eye of a 35-year-old patient with chronic RD and phthisis bulbi. (**B, D, F, H**) Show slides from the two autopsy eyes of older patients with age-related drusen. Both drusen groups stained positively with PAS **A** and **B**, negatively with LFB **C** and **D** and Von Kossa **G** and **H**, and blue on Masson Trichrome (**E** and **F**). Focal dystrophic calcification within a druse is highlighted with Von Kossa **G** (*red arrow*). No calcifications were seen in the control eyes **H**. *Red/black asterisk* = drusen; *black arrows* = Bruch's membrane; *green arrows* = retinal pigment epithelium; and *red arrow* = calcifications.

Drusen in the evisceration specimen had significantly larger average circumference (278 vs. 52 µm, *P* < 0.001, Cohen's ds = 2.55), largest diameter (96 vs. 20 µm, *P* < 0.001, Cohen's ds = 2.68), and area (0.006 vs 0.0002 mm^2^, *P* < 0.001, Cohen's ds = 1.82) than age-related drusen, respectively (see the Table). Both groups displayed positive staining with PAS (Figs. 3A, 3B), negative staining with LFB (Figs. 3C, 3D), and stained blue with Masson Trichrome (Figs. 3E, 3F). Von Kossa highlighted dystrophic calcification seen in a subset of the large drusen deposits in the eviscerated specimen (Fig. 3G) but not in the control eyes (Fig. 3H).

## Discussion

In this study, we found significant differences in morphometric parameters of atypical drusen in the eye of a young patient with long-standing RD compared to age-related drusen. The eviscerated specimen displayed significantly larger drusen in diameter, circumference, and area than the age-related drusen in autopsy eyes of older patients. These atypical drusen had a spheroidal shape with a radiating fibrillar pattern in contrast to the dome-shaped appearance of age-related drusen in controls. Both drusen groups had a similar histochemical staining pattern, which may suggest a similar macromolecule composition, including polysaccharides (PAS positive) and collagenous material (blue staining on Masson Trichrome). Despite the overall negative staining with Van Kossa, focal areas of dystrophic calcification were noted within some of the atypical drusen in the evisceration specimen but not age-related drusen.

Drusen are commonly found in younger adults, with a reported prevalence ranging between 30% in adults aged 20 to 24 and 49% to 91% in those aged 45 to 54 based on digital retinal images.^8^^,^^9^ Small hard drusen (<63 µm) are quite common in younger patients.^13^ Large drusen (>125 µm), on the other hand, are rarely seen in younger adults (0 to 1.2% population),^8^^,^^9^ but the prevalences increase with age: from 2 per 100 persons for ages 40 to 49, to 2.95 to 3.97 for ages 50 to 59, to 5.41 to 7.43 for ages 60 to 69, to 10.23 to 14.07 for ages 70 to 79, and 23.56 for those over 80 years.^14^

The exact pathogenesis of drusen formation is yet to be elucidated. They are composed of various acellular debris, carbohydrates, cholesterol, and lipoproteins. Immunoglobulin light chains, amyloid, and complement proteins^15^^,^^16^ have also been identified in drusen, suggesting that these deposits might form as a response to local or systemic inflammation.^16^^–^^18^ Dysfunction of the complement system proteins is thought to be a major contributor to drusen formation and progression to geographic atrophy,^19^ and complement proteins were identified as effective targets in the treatment of AMD and led to two US Food and Drug Administration drug approvals,^19^ pegcetacoplan^20^ and avacincaptad pegol.^21^ Pro-inflammatory markers have also been correlated with the presence of drusen in eyes, particularly in younger patients.^22^ Our eviscerated specimen had evidence of chronic inflammation within the choroid and within some drusen, suggesting a possible contribution of inflammation to drusen formation. Additionally, excessive formation of reactive oxygen species (ROS) has been recognized as a key contributor to drusen formation in AMD and implicated in other retinal pathologies.^23^^,^^24^ Huang et al. showed that eyes with RD have significantly higher concentrations of ROS compared with controls^25^; thus, it is possible that long-standing RD generates a chronic low-grade inflammatory environment, favorable to drusen formation.

Genetics are also thought to play a substantial role in drusen pathophysiology. Concordant twin studies have identified the presence of soft drusen or more than 20 hard drusen as highly heritable features.^13^^,^^26^^,^^27^ With over 20 drusen identified in our eviscerated specimen, it is possible that some heritable features may also account for the origin of these drusen. Considering the rarity of large drusen in a young population and their atypical appearance, it is unlikely that the large drusen observed in our young patient are naturally occurring and may instead represent a sequela of inflammation and have some hereditary component. However, the present study did not assess these molecular features and such interpretations remain hypothetical.

To date, we have identified 2 similar case reports of large drusen found in the enucleated eyes of two 23-year-old women, both with a history of childhood eye injuries and chronic RDs.^10^^,^^11^ Raman et al. and Shenoy et al. also reported large (0.2 mm) spherical drusen with radiating fibrils that had a similar composition as our atypical drusen based on the histochemical staining pattern (PAS positive, blue staining on Masson Trichrome).^10^^,^^11^ In contrast to our case, the drusen in Shenoy et al.’s case demonstrated positive staining with LFB, suggesting the presence of lipoprotein within the drusen.^10^ However, our negative staining results with LFB should be interpreted cautiously, as the stain is subject to frequent false negative results and may not be a reliable marker for the detection of lipoprotein. Soft drusen tend to have a higher esterified cholesterol than lipoprotein,^28^ and immunolabeling for apolipoproteins A, B, and E, as done in prior AMD studies,^28^^,^^29^ may thus be a more accurate method for evaluating drusen composition in future investigations.

Additionally, Shenoy et al. noted instances of focal osseous metaplasia and focal microcalcification within some drusen,^10^ but the Von Kossa stain was not performed in either study.^10^^,^^11^ Calcified drusen have also been reported by Mitsuma et al. in an eye of a young man with chronic RD after silicone oil removal.^30^ Knorr et al. showed that intraretinal vacuoles can be found in the retina 4 weeks after instillation,^31^ and it is likely sequestered due to persistent RD or retinal trauma.^32^ Several studies have documented an inflammatory response associated with silicone oil injection^31^^–^^34^ that can manifest as surrounding inflammatory cells and pigment granules.^32^ No pigment granules were visualized surrounding the vacuoles in our eviscerated specimen, but the identified areas of chronic inflammation in the choroid and within some drusen suggest that they could potentially form as a sequela of inflammation secondary to silicone oil placement.

These cases^10^^,^^11^^,^^30^ suggest a possible pattern of drusen formation in association with chronic RD. Generally, drusen are not known to form in response to RD; rather, several cases reported on temporal regression of drusen after retinal detachment and macular hole repairs.^35^^–^^39^ The mechanism for this phenomenon is not known, but some theories include the removal of vitreous and ensuing mechanism clearance of drusen during pars plana vitrectomy^39^ and laser coagulation-induced phagocytosis of drusen debris by macrophages and RPE cells.^40^ Future large investigations on the temporal association of RD and drusen formation, particularly in younger individuals.

Overall, the similarities in clinical presentation and histomorphometric features with Raman et al. and Shenoy et al.’s cases,^10^^,^^11^ suggest that these atypical drusen may be associated with younger age, female sex, chronic RD, or eye trauma. It remains unclear whether these atypical drusen represent a distinct pathological entity or a variation of age-related drusen. Notably, these drusen were described as “giant drusen” by Shenoy et al. and Raman et al.,^10^^,^^11^ but this term has to be distinguished from “giant drusen” of the optic nerve in astrocytic hamartoma. Additionally, large drusen in young adults may be confused with AMD. Although rare, the Beaver Dam Offspring Study estimated the prevalence of early AMD is between 2.1% and 2.4% in adults under 45 years of age.^41^ In the absence of histopathological samples, patient history and clinical examination findings of long-standing RD would be less suggestive of AMD.

The small number of eyes included in the study is naturally a limitation of the study. It is important to note that these observations apply primarily to the present case and cannot be extrapolated to all patients with chronic RD, given that only one young eye with atypical drusen was included in the study. Furthermore, no fundus or OCT images were available for the eyes examined in this study in order to correlate the histologic appearance with clinical examination. Most published studies rely on drusen size classification based on OCT measurements. Our study presented two-dimensional drusen measurements using formalin-fixed, paraffin-embedded specimens, making comparison with OCT measurements less reliable. Additionally, the intra- and inter-observer repeatability of the histomorphometric parameters has not been evaluated and thus may be a source of error. Nevertheless, this study is one of the very few reports of histopathological examination of large drusen in young patients. Further investigations are warranted to elucidate the natural history of these atypical drusen and explore their association with RD, age, and sex. Larger case series are advised for future investigations, and the studies may benefit from advanced molecular analysis and imaging-histology to better characterize histopathology and nature history of these drusen.
